# Characterization of *Cutibacterium acnes* isolated from prosthetic joint infections and healthy skin

**DOI:** 10.3389/fsurg.2026.1901396

**Published:** 2026-09-09

**Authors:** Elena De Vecchi, Marta Bottagisio, Alessandro Bidossi, Francesca Villa, Nicola Logoluso, Luigi Zagra

**Affiliations:** 1 Laboratory of Clinical Chemistry and Microbiology, IRCCS Ospedale Galeazzi Sant’Ambrogio, Milan, Italy; 2 C.R.I.O. Unit, IRCCS Ospedale Galeazzi Sant’Ambrogio, Milan, Italy; 3 Hip Department IRCCS Istituto Ortopedico Galeazzi, Milan, Italy; 4 Università Vita-Salute San Raffaele, Milan, Italy

**Keywords:** bacterial aggregates, bacterial biofilm, *Cutibacterium acnes*, diagnosis of implant-related infections, prosthetic joint infections

## Abstract

*Cutibacterium acnes* is an anaerobic bacterium that contributes to the balance of the skin microbiota but is also responsible for prosthetic joint infections (PJIs). Phenotypic and genotypic characterization of *C. acnes* isolated from infected implants could identify specific traits associated with a higher risk of PJI. To address this hypothesis, the phenotypic and genotypic traits of 20 clinical isolates were analyzed and compared with those of seven strains isolated from the skin microbiota of healthy participants. In particular, the antibiotic susceptibility profile and the ability of bacteria to aggregate and form biofilms *in vitro* were investigated. In addition, the genomes of all isolates were sequenced to determine whether PJI-causing microorganisms share a distinctive phylogenetic profile. Antibiotic susceptibility, biofilm formation, and aggregate formation did not differ between commensal and pathogen isolates. By contrast, extracellular DNA was mostly present in clinical isolates compared with commensal *C. acnes*. Phylogenetic analysis failed to evidence differences between pathogens and commensal strains, while a phylogenetic distance was observed between sequence types. Results suggest that *C. acnes* isolated from PJIs and healthy skin share common phenotypic and genotypic traits, making them indistinguishable. Hence, further studies on the phenotypic and genotypic characteristics of *C. acnes* are needed, as the association between diverse phylotypes and diseases remains unclear and debated.

## 1 Introduction

Prosthetic joint arthroplasties (PJAs) are widely performed orthopedic procedures and effective therapeutic options for symptomatic osteoarthritis associated with functional limitation and pain, as well as for managing post-traumatic events. Undeniably, PJAs have high success rates in providing long-term pain relief and restoring joint function (1–3). As the population ages, the incidence of total hip and knee arthroplasties is projected to increase in alignment with this global trend. Prosthetic joint infections (PJIs) are one of the major causes of implant failure, requiring replacement of the prosthetic elements and having a burdensome impact on patient quality of life and hospitalization costs (4).

*Cutibacterium acnes* is a Gram-positive, anaerobic, slow-growing, rod-shaped bacterium physiologically present on the skin. The high prevalence of *C. acnes* on the skin of healthy participants poses a risk when an orthopedic device is implanted (5). If contamination occurs, the presence of an abiotic surface on which opportunistic bacteria can adhere and form biofilms represents an important risk factor. Furthermore, the documented ability of *C. acnes* to secrete extracellular polymeric substances (EPS) onto the surfaces of implanted biomaterials results in the development of a difficult-to-eradicate prosthetic joint infection (6). *C. acnes*-mediated PJIs represent a clinical challenge, being difficult to diagnose while still requiring prompt and adequate management. Indeed, they often present no clear signs and symptoms, as in acute implant-related infections, only resulting in a painful mobilization. Multiple studies have investigated risk factors for *C. acnes* in shoulder infections, commonly reporting male sex, presence of hair, shoulder steroid injections, and previous shoulder surgery (7). Its clinical presentation is often non-specific and can occur long after arthroplasty, leading to delayed diagnosis. Long-term follow-up of *C. acnes* cases has revealed osteolysis around shoulder implants, and the microbial ability to invade the osteocyte lacuno-canalicular networks within bone has been demonstrated in an implant-associated osteomyelitis murine model (8). *C. acnes* has been widely studied, and its genome has been characterized to define evolutionary lineages according to their allelic variants. In particular, McDowell and colleagues defined four distinct phylotypes, IA, IB, II, and III, based on the multilocus sequence type (MLST) profile of *C. acnes* isolates (9), and it has been suggested that *C. acnes* lineage might be associated with the probability of developing a specific disease (10, 11).

In such a complex scenario, the characterization of the microorganisms isolated from implants and periprosthetic tissues might identify specific particular phenotypes or genotypes associated with a higher risk of developing PJI or indicate whether all isolates can potentially mediate an implant-related infection under favorable conditions. Furthermore, the characterization of the genomes of *C. acnes* isolates might reveal genetic hallmarks of these bacteria, supporting and expediting the challenging diagnostic process.

To address this experimental hypothesis, the phenotypic and genotypic traits of 20 *C. acnes* clinical isolates were analyzed and compared with those of seven *C. acnes* isolates from the skin of healthy participants. In particular, the antibiotic susceptibility profile and the ability of bacteria to aggregate and form biofilms *in vitro* were investigated. In addition, the genomes of all tested isolates were sequenced to accurately classify bacterial species and to determine whether PJI-causing microorganisms share a distinctive phylogenetic profile.

## 2 Materials and methods

### 2.1 Sample collection and growth conditions

Twenty clinical isolates of *C. acnes* recovered from periprosthetic tissue or medical device systems explanted from patients admitted to the IRCCS Ospedale Galeazzi–Sant'Ambrogio who fulfilled the diagnostic criteria for PJI were evaluated (12). These strains were isolated during routine microbiological diagnostic workflows for PJI and stored in our collection. Briefly, at least five samples, including biotic and abiotic explanted material, were sent to the Laboratory of Clinical Chemistry and Microbiology of the hospital immediately following surgical procedures. There, the specimens were treated with a 0.1% dithiothreitol (DTT, Sigma-Aldrich) solution for 15 min under agitation to dislodge bacterial cells attached to the implant surface (13). The DTT solution was then collected and centrifuged for 15 min, and the pellet was suspended in 1 mL of sterile saline solution. Finally, 100 μL was either plated onto agar plates (chocolate agar, MacConkey agar, mannitol salt agar, and Sabouraud agar) or inoculated into 10 mL of brain heart infusion (BHI) and thioglycollate broth (THB). Agar plates were incubated for 48 h at 37° C in aerobic conditions or in a 10% CO_2_-enriched atmosphere, and broth tubes were cultured at 37°C for up to 15 days. *C. acnes* strains were isolated from THB showing turbidity, which were then plated onto Schaedler agar plates and incubated in an anaerobic environment.

Similarly, seven *C. acnes* strains were isolated from the skin of laboratory workers without any known dermatologic conditions, who were considered healthy controls. In particular, commensal *C. acnes* were recovered from the volunteers' head using a sterile cotton swab streaked onto Schaedler agar plates and cultured for 48 h in an anaerobic environment before characterization.

The identification of isolates was carried out using the automated VITEK2 system (bioMérieux) and the ANC ID card (bioMérieux), specifically designed for the identification of anaerobic and coryneform bacteria at the species level. The susceptibilities to benzylpenicillin, meropenem, clindamycin, tetracycline, erythromycin, imipenem, vancomycin, and ampicillin were investigated by the epsilometer test (ETEST, bioMérieux). Briefly, the epsilometer test strips were placed on Mueller–Hinton fastidious agar plates enriched with 5% horse blood (MH-F, bioMérieux), inoculated with a 3 McFarland bacterial suspension, and incubated at 37°C under anaerobic conditions for 48 h.

### 2.2 Evaluation of biofilm biomass formation

The biofilm biomass production was evaluated according to the spectrophotometric assay proposed by Christensen and colleagues (14). Briefly, *C. acnes* strains were grown on Schaedler agar plates under anaerobic conditions for 48 h. Subsequently, 20 μL of 0.5 McFarland suspension was inoculated with 180 μL of BHI broth under anaerobic conditions. Two hundred microliters of BHI without bacterial inoculum were used as negative controls. Four assays were run in triplicate for each tested strain (*n* = 12, per isolate). At the end of a 72 h incubation, wells were washed three times with sterile 0.9% NaCl solution to remove all biological components except the biofilm. After the final wash step, the 96-well plates were dried, and the biofilm biomass was stained with 200 μL of 0.1% crystal violet (CV) solution for 10 min at room temperature. The wells were washed thrice with sterile saline solution to remove excess stain. Once completely dry, 200 μL of absolute ethanol was added to each well to solubilize the dye bound to the biofilm biomass. To quantify the extracted CV, the optical density (OD) was read at 595 nm by using a microplate reader (Multiskan FC, Thermo Scientific, Italy). Isolates were classified as strong, moderate, or weak biofilm producers, according to criteria proposed by Stepanovic et al. (15).

### 2.3 Evaluation of bacterial aggregation in synovial fluid

The ability of *C. acnes* isolates to aggregate *in vitro* in biological fluids was investigated. Briefly, colonies from Schaedler agar plates cultured under anaerobic conditions for at least 48 h were suspended in sterile saline solution to a turbidity of 0.5 McFarland and inoculated into bovine synovial fluid (BSF, Lampire Biological Laboratories, Inc.) in a 1:10 ratio. After a 24-h culture at 37°C with agitation at 200 rpm, 10 µL of the suspension was placed on a microscope glass slide (SuperFrost, Thermo Scientific), fixed with 4% paraformaldehyde (Carlo Erba), and dried on a hot plate at 50°C. Samples were then stained with CV solution (Carlo Erba), analyzed by an upright optical microscope with a 40× objective (CX43 Olympus), and finally quantified with Fiji software (Fiji, ImageJ, Wayne Rasband, National Institutes of Health). Three drops from each sample were analyzed, and three independent experiments were carried out (*n* = 9, per strain).

### 2.4 Confocal microscopy analysis of biofilm-like aggregates

The composition of the matrix separated by the bacterial aggregates was analyzed using an upright TCS SP8 (Leica Microsystems CMS GmbH) CLSM, following a validated protocol established by our research group (16).

Similar to the previously described aggregation assays, *C. acnes* cells were cultured at 37°C on a shaker at 200 rpm in BSF (Lampire Biological Laboratories, Inc.) under anaerobic conditions. After a 48-h culture, 10 µL of supernatants was gently collected and incubated for 30 min in the dark with either (1) a 0.5 mM SYTO 9 green fluorescent nucleic acid stain solution (Thermo Fisher Diagnostics), (2) a 5 µg/mL wheat-germ agglutinin (WGA) Alexa Fluor 647 conjugate (Invitrogen), or (3) 50 µg/mL α-fibrin alpha chain antibody conjugated to Cy5 (Abcore, Inc.) in sterile deionized water. Thereafter, samples were gently streaked over the surface of a glass slide, air-dried, and then observed. Images were acquired using a 20× dry objective (HC PL FLUOTAR 20/0.50 DRY) and a 100× immersion oil objective (HC PL APO 100X/1.40 OIL CS2). The images obtained were processed using Las×(Leica Microsystems CMS GmbH).

### 2.5 Whole-genome sequencing

To explore the genomic differences between pathogenic and commensal *C. acnes* strains, bacterial pellets were collected and sent to MicrobesNG (http://www.microbesng.com) for sequencing. An isolated colony from each isolate was streaked onto Schaedler agar plates and cultured under anaerobic conditions for approximately 48 h. Thereafter, bacterial cultures were collected and suspended in 10 mL of sterile phosphate-buffered saline (PBS). The OD was then measured at 600 nm using a microplate reader (Multiskan FC, Thermo Scientific, Italy). An equivalent of 8–12 OD at 600 nm corresponding to 4–6 × 10^9^ cells was centrifuged at 3,000*g* for 10 min at 4°C, and the bacterial pellet was washed twice with ice-cold PBS. Finally, the bacterial pellet was dissolved in DNA/RNA Shield (Zymo Research) and transferred to barcoded tubes for shipment. At the microbial sequencing service facility, 5–40 μL of bacterial suspension was lysed in 120 μL of Tris-EDTA buffer solution containing 0.1 mg/mL lysozyme and 0.1 mg/mL RNase A (ITW Reagents). Lysed samples were then incubated for 25 min at 37°C. Thereafter, 0.1 mg/mL proteinase K (VWR Chemicals, Ohio, USA) and 0.5% v/v sodium dodecyl sulfate (Sigma-Aldrich) were added, and the mixture was incubated for 5 min at 65°C. Genomic DNA was purified using an equal volume of SPRI beads and resuspended in an elution buffer (Qiagen, Germany). Finally, DNA was quantified using the Quant-iT dsDNA HS kit (Thermo Fisher Scientific) assay in an Eppendorf AF2200 plate reader (Eppendorf UK Ltd., United Kingdom). Whole-genome sequencing was performed using Illumina technology. Genomic DNA libraries were prepared using the Nextera XT Library Sample Preparation Kit (Illumina, San Diego, USA) following the manufacturer's protocol except for the following modifications: a twofold increase in input DNA and a 45 s increase in PCR elongation time. DNA quantification and library preparation were performed using a Hamilton Microlab STAR automated liquid-handling system (Hamilton Bonaduz AG, Switzerland). Thereafter, pooled libraries were quantified using the Kapa Biosystems Library Quantification Kit for Illumina and sequenced using Illumina sequencers (HiSeq/NovaSeq) following the 250-bp paired-end protocol. Quality filtering was performed using Trimmomatic (v0.30) with a sliding-window quality cutoff of Q15 (17). *De novo* assembly was performed using the SPAdes assembler (v3.7) (18), and contigs were annotated using Prokka (V1.11) (19).

#### 2.5.1 Bioinformatic analyses

Sequence analyses to define MLST profiles of *C. acnes* clinical and commensal isolates were performed on the PubMLST website (https://www.pubmlst.org). Briefly, FASTA files were loaded and queried against databases to define the allelic profile through a gene-by-gene analysis of partial sequences from six-core housekeeping genes—*aro*E (shikimate 5-dehydrogenase), *atp*D (ATP synthase beta chain), *gmk* (guanylate kinase), *gua*A (GMP synthase), *lep*A (GTP-binding protein), and *sod*A (superoxide dismutase)—and complete sequences from two putative virulence genes—*tly* (putative hemolysin) and *camp*2 (Christie Atkins Munch Petersen, CAMP homolog) (10, 20). In doing so, the online tool automatically assigned integer numbers to each unique sequence analyzed by strain and defined the sequence type (ST) for each unique combination of allelic integers. Likewise, BURST, GrapeTree, and interactive Tree of Life (iTOL) analyses were performed on PubMLST.org. The BURST algorithm was used to group MLST-type data based on the count of profiles that match at specified numbers of loci. Similarly, using GrapeTree and iTOL plugins, phylogenetic trees were generated to graphically visualize the evolutionary descent lineages of different *C. acnes* strains. All the aforementioned PubMLST plugins used were enabled by the Bacterial Isolate Genome Sequence database (BigsDB) developed by Jolley and colleagues (21). Finally, whole-genome sequences of *C. acnes* clinical strains were submitted and deposited in the DBJ/ENA/GenBank public repository, and MLST schemes were also submitted and deposited in the PubMLST.org databases.

### 2.6 Statistical analysis

Statistical analyses were performed using GraphPad Prism version 8.0.2 (GraphPad Software, San Diego, CA, USA). Technical replicates were averaged to obtain a single biological value for each isolate, and each isolate was treated as the experimental unit in all statistical analyses. Data distribution was assessed using the Shapiro–Wilk normality test. To compare biomass formation between clinical and commensal isolates, the Mann–Whytney *U*-test was used, while the comparison of the same outcome among the three phenotypes Ia, Ib, and II was performed using a Kruskal–Wallis test with Dunn's multiple comparisons. Two-way ANOVA with Tukey's multiple comparisons test was used to analyze bacterial aggregates of different sizes across the total area occupied by bacteria in clinical and commensal isolates. The Fisher exact test was used to analyze the percentage of isolates classified as having strong, moderate, or weak ability to form biomass, to analyze the percentage of clinical and commensal isolates showing WGA and BOBO-3 signals, and to compare the percentage of Ia, Ib, and II phenotypes between clinical and commensal isolates. The Fisher exact test was performed in R software version 4.6.1. All statistical tests were two-tailed, and *p*-values <0.05 were considered statistically significant.

## 3 Results

### 3.1 Antibiotic susceptibility

Isolates were identified as *C. acnes* by the VITEK® 2 system (bioMérieux) and then confirmed by sequencing analysis. The antibiotic susceptibility profile to a panel of eight antibiotics was tested using the E-test (ETEST®, bioMérieux), and results are summarized in Table 1. The analyzed *C. acnes* isolates showed widespread susceptibility to all tested antibiotics, with only sporadic exceptions. Among the tested molecules, erythromycin and clindamycin were less effective therapeutic options for *C. acnes* 10 and C_07. No differences were detected between pathogens and commensal strains, despite the common misconception that PJI-mediating bacteria possess resistance traits and that commensal strains lack them.

**Table 1:** Antibiotic susceptibility profile of *C. acnes* isolates.

ID	BEN	TETR	MERO	VANCO	CLIND	ERIT	IMIP	AMPI
01	0.016	0.016	0.064	0.50	0.023	0.016	0.012	0.047
02	0.004	0.047	0.012	0.25	0.023	0.023	0.004	0.016
03	0.006	0.047	0.032	0.38	0.023	0.016	0.004	0.016
04	0.006	0.016	0.016	0.38	0.016	0.016	0.004	0.016
05	0.023	0.047	0.064	0.25	0.023	0.016	0.012	0.047
06	0.003	0.016	0.004	0.19	0.023	0.016	0.002	0.016
07	0.006	0.016	0.016	0.38	0.016	0.032	0.004	0.016
08	0.006	0.032	0.016	0.25	0.023	0.016	0.008	0.032
09	0.006	0.032	0.008	0.25	0.023	0.032	0.008	0.032
10	0.006	0.032	0.008	0.25	256	256	0.006	0.032
11	0.012	0.047	0.012	0.25	0.032	0.016	0.008	0.032
12	0.006	0.016	0.016	0.38	0.016	0.023	0.006	0.016
13	0.012	0.023	0.032	0.38	0.016	0.016	0.008	0.016
14	0.004	0.047	0.006	0.25	0.023	0.016	0.004	0.016
15	0.012	0.032	0.032	0.50	0.023	0.016	0.008	0.016
16	0.004	0.016	0.008	0.50	0.016	0.016	0.006	0.016
17	0.006	0.047	0.008	0.38	0.023	0.016	0.008	0.023
18	0.012	0.016	0.023	0.38	0.023	0.016	0.006	0.016
19	0.006	0.016	0.006	0.016	0.023	0.023	0.003	0.016
20	0.004	0.016	0.012	0.016	0.023	0.023	0.006	0.016
C_01	0.006	0.047	0.008	0.38	0.023	0.016	0.004	0.016
C_02	0.003	0.016	0.002	0.50	0.016	0.016	0.002	0.016
C_03	0.006	0.064	0.004	0.38	0.023	0.047	0.012	0.032
C_04	0.004	0.032	0.006	0.38	0.032	0.023	0.003	0.016
C_05	0.004	0.047	0.016	0.38	0.016	0.016	0.004	0.016
C_06	0.004	0.125	0.006	0.50	1.5	256	0.003	0.016
C_07	0.016	0.047	0.047	0.50	0.047	0.016	0.016	0.016

MIC values are expressed as µg/mL. BEN: Benzylpenicillin, TETR: Tetracycline, MERO: Meropenem, VANCO: Vancomycin, CLIND: Clindamycin, ERIT: Erithromycin, IMIP: Imipenem, AMPI: Ampicillin.

### 3.2 Evaluation of biofilm biomass formation

**Figure 1:**
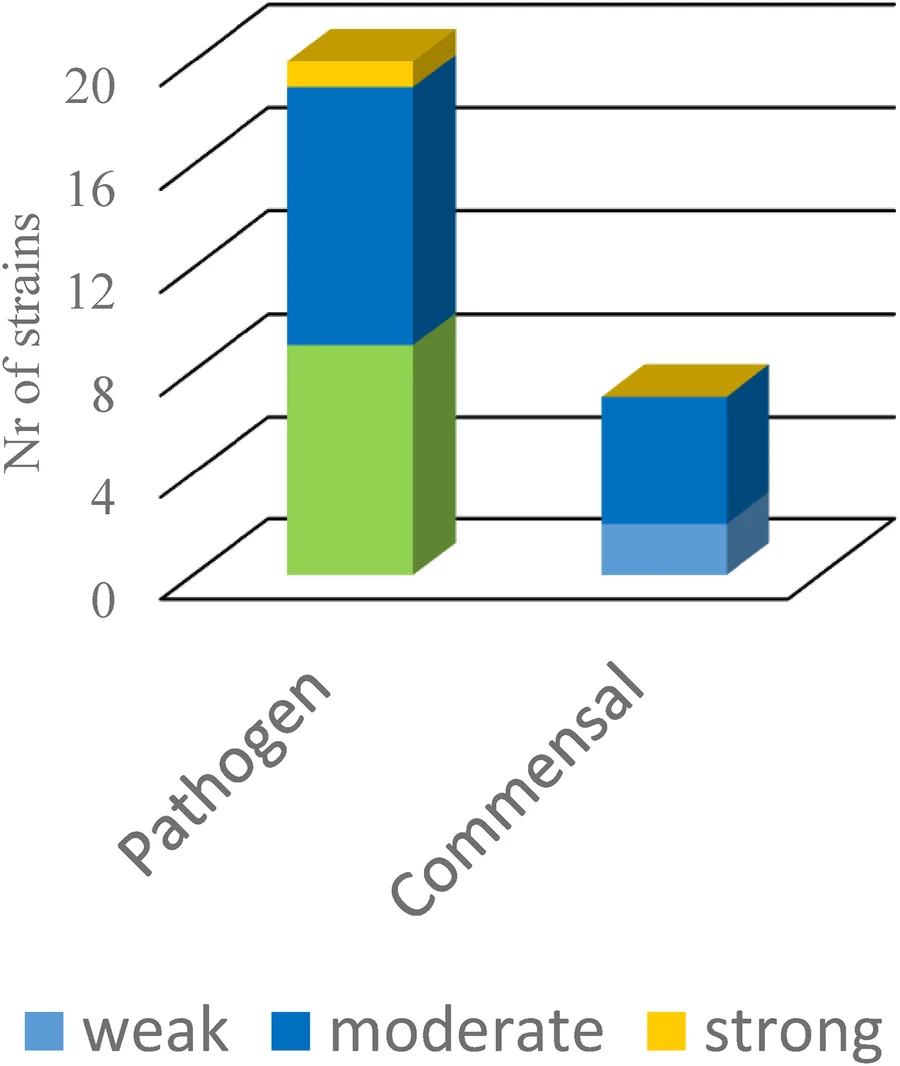
Classification of analyzed strains according to their biofilm production *in vitro*. Green: weak biofilm producers; blue: moderate biofilm producers; and yellow: strong biofilm producers.

The ability to produce EPS was assessed by quantifying the separated biofilm biomass via CV spectrophotometry. Absorbance values were evaluated to classify *C. acnes* pathogens and commensal isolates into strong, moderate, weak, or non-biofilm producers (Figure 1). None of the analyzed *C. acnes* isolates was a non-biofilm producer. All the tested strains demonstrated the ability to secrete EPS in the tested *in vitro* conditions. However, no difference between the absorbance values of clinical isolates (1.17 ± 0.07) and those of commensal strains (1.16 ± 0.03) was observed (*p*-value = 0.756). In particular, 50% of the clinical isolates were classified as moderate biofilm producers, 45% as weak, and only one isolate (5%) as a strong biofilm producer. Interestingly, none of the commensal strains isolated from healthy participants were classified as strong EPS producers; however, 70% were moderate biofilm producers. However, no statistically significant differences between clinical and commensal isolates in biofilm biomass formation were observed (*p*-value = 0.746).

### 3.3 Evaluation of bacterial aggregation in synovial fluid

**Figure 2:**
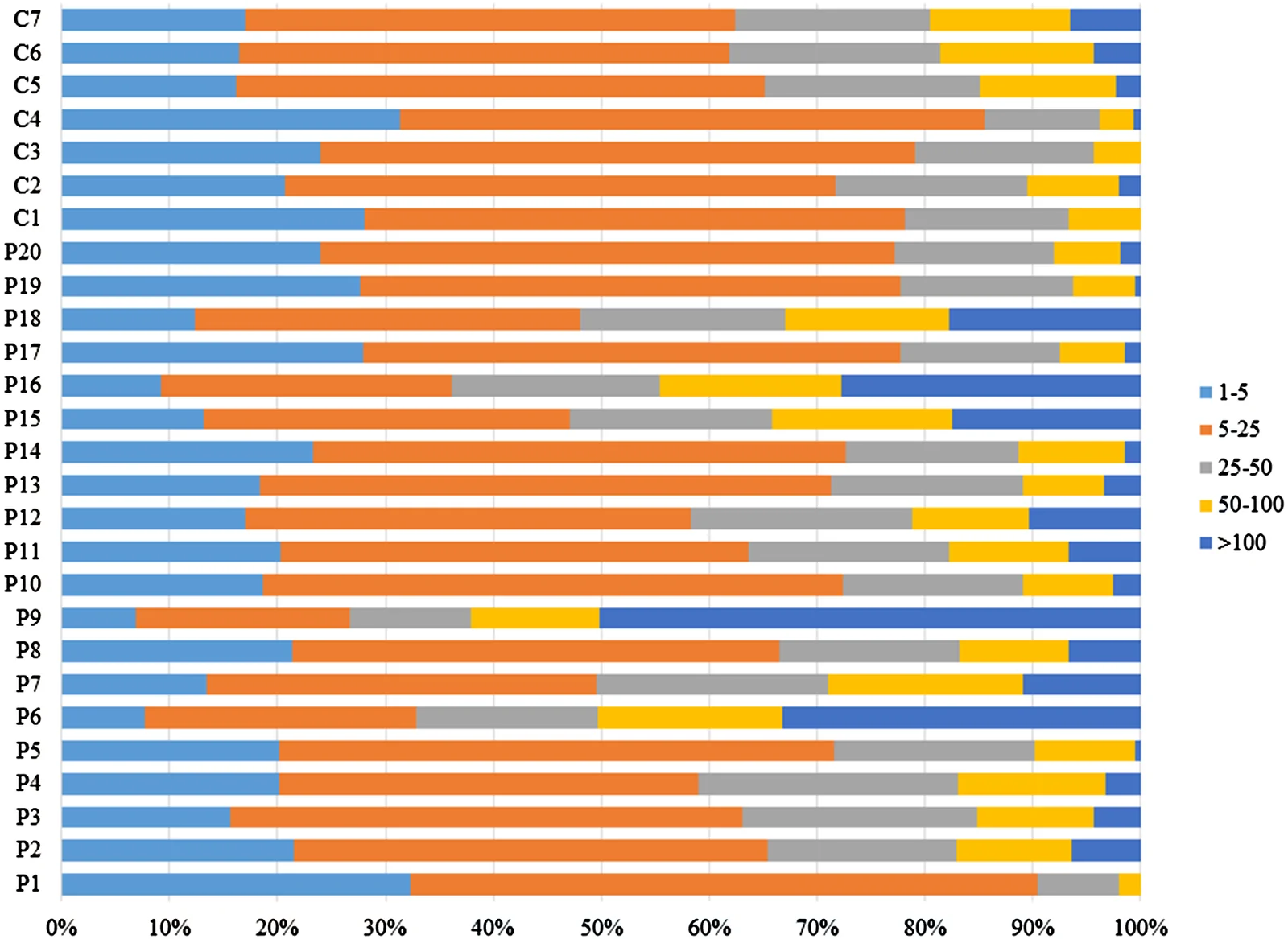
Graphical representation of the amount (%) of *C. acnes* aggregates per size (µm^2^) on the total surface area analyzed.

The behavior of bacteria in biological fluids was investigated by evaluating the ability of *C. acnes* to form free-floating aggregates when cultured in synovial fluid. The surface area of bacterial aggregates was quantified using Fiji software and graphically reported as the percentage of the aggregate surface area relative to the total area occupied by bacteria per sample (Figure 2). Only three isolates (pathogen 01 and commensals C_01 and C_03) were unable to form aggregates with a surface area >100 μm^2^. A significant effect of aggregate surface distribution for both pathogenic and commensal isolates was observed (effect size=0.736; *p* < 0.0001); on average, most of the aggregates fell within the size range of 5–25 μm^2^ in all the samples analyzed, regardless of the origin (PJIs vs. healthy skin microbiota). Nevertheless, pathogenic isolates formed significantly larger aggregates than commensal isolates (effect size = 0.048, *p*-value < 0.0001). Indeed, the data reported in Figure 2 are expressed as the percentage of different-sized aggregates relative to the total surface area occupied by bacterial cells. Consequently, a high number of planktonic cells and small bacterial aggregates might appear as an unimportant minor fraction compared to the stronger signal of mounds measuring >100 μm^2^ detected and reported in our series.

### 3.4 Confocal microscopy analysis of biofilm-like aggregates

**Figure 3:**
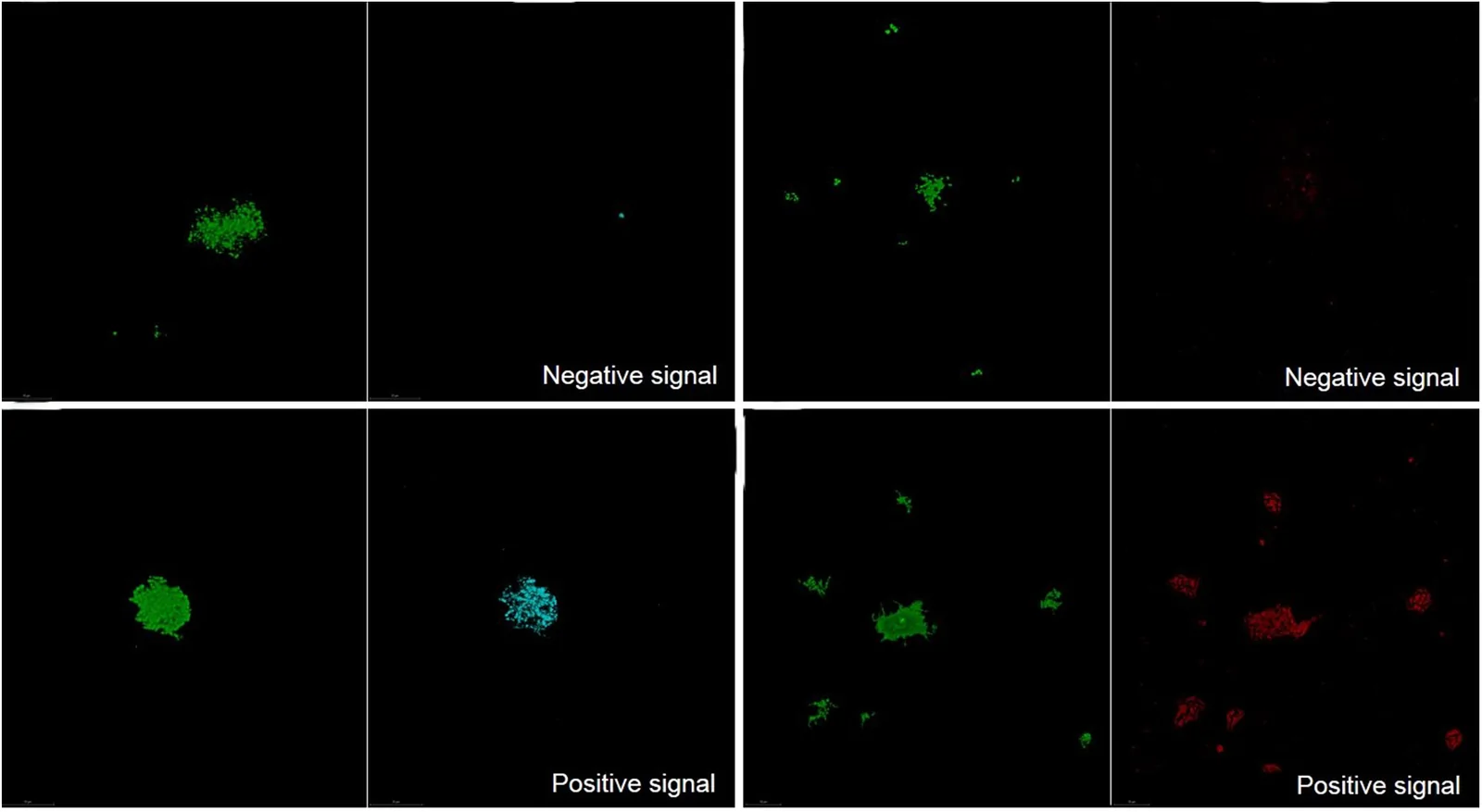
Representative CLSM images of *C. acnes* aggregates. Cellular biomass was stained with SYTO 9 (green signal), eDNA was stained with BOBO-3 (light blue signal), and GlcNAc-containing polysaccharides were stained with WGA dye (red signal).

The biofilm-like aggregates grown in synovial fluid were also qualitatively analyzed using CLSM, and the representative micrographs are reported in Figure 3.

The signal of WGA-stained *N*-acetylglucosamine and BOBO-3-stained eDNA localized closely with that of SYTO 9, showing an intensity directly proportional to the size of the aggregates.

WGA signal was not detected in only two bacterial isolates (one infecting and one commensal). Conversely, the BOBO-3 signal for eDNA detection was absent in the 60% of analyzed commensal *C. acnes*, while it was present in 80% of the clinical isolates. However, no significant differences were observed in either signal between bacterial isolates and commensals (BOBO-3-stained eDNA *p*-value = 0.328; WGA *p*-value = 0.444).

### 3.5 Whole-genome sequencing

The genome of all isolates was sequenced, and the results are summarized in Table 2.

**Table 2:** Whole-genome sequencing results obtained from the analysis of 27 *C. acnes* isolates.

ID	GeneBank	Total length (bp)	Contigs	N50 (bp)
01	SAMN33482185	2,545,072	14	646,800
02	SAMN33482186	2,535,310	8	893,106
03	SAMN33482187	2,534,888	11	700,755
04	SAMN33482188	2,547,635	12	362,919
05	SAMN33482189	2,544,747	12	572,712
06	SAMN33482190	2,479,065	10	627,814
07	SAMN33482191	2,476,990	9	627,777
08	SAMN33482192	2,482,215	10	884,059
09	SAMN33482193	4,457,039	1,315	9,954
10	SAMN33482194	2,484,608	21	181,117
11	SAMN33482195	2,479,637	11	820,736
12	SAMN33482196	2,536,531	9	893,633
13	SAMN33482197	2,546,857	16	337,178
14	SAMN33482198	2,537,259	15	340,148
15	SAMN33482199	2,536,534	9	893,465
16	SAMN33482200	2,535,886	9	893,465
17	SAMN33482201	2,546,084	8	908,970
18	SAMN33482202	2,547,319	10	646,675
19	SAMN33482203	2,473,011	11	627,839
20	SAMN33482204	2,546,314	9	908,963
C_01	SAMN33482206	2,542,684	15	821,319
C_02	SAMN33482207	2,479,176	11	627,735
C_03	SAMN33482208	2,960,772	531	893,014
C_04	SAMN33482209	2,533,738	16	779,931
C_05	SAMN33482210	2,478,730	8	892,995
C_06	SAMN33482211	2,477,695	11	828,423
C_07	SAMN33482213	2,474,927	19	392,221

#### 3.5.1 Multilocus sequence typing

**Figure 4:**
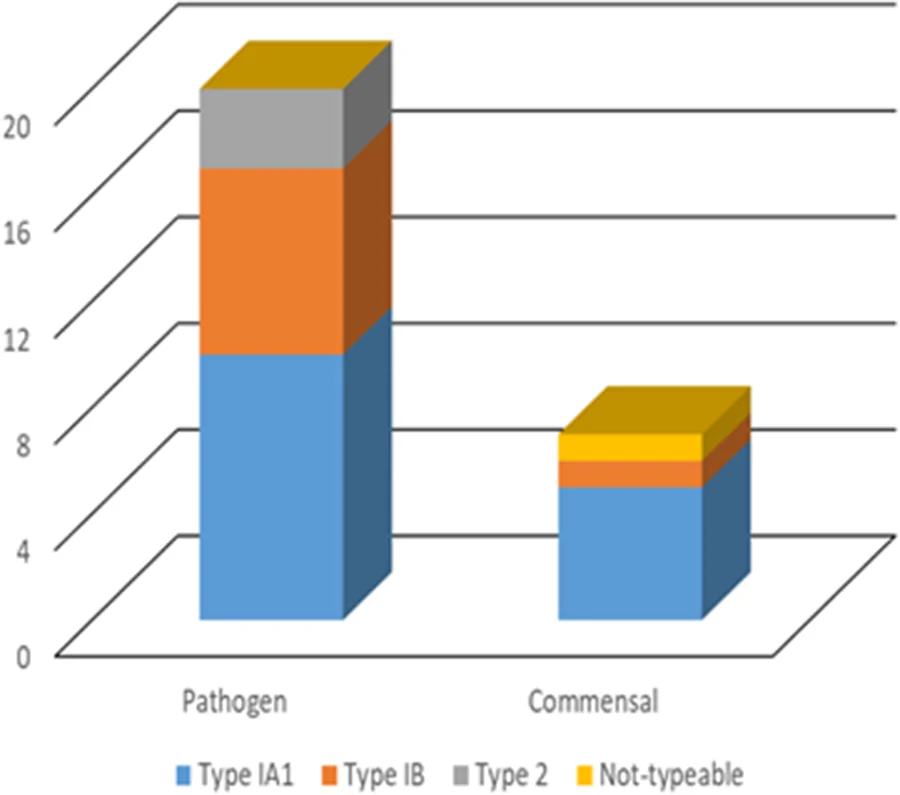
Distribution of the MLST type in commensal and pathogen *C. acnes*.

The phylotype of *C. acnes* was defined by querying whole-genome data on PubMLST.org. In particular, alleles of the defining loci, together with the ST, the clonal complex (CC), and the evolutionary lineage (types IA, IB, II, and III) were determined, as reported in Table 3. From the *in silico* analysis, the commensal *C. acnes* C_09 was classified as ST 105 (ST linking CC1 and CC5), and no evolutionary lineage could be assigned. Therefore, it was excluded from subsequent analyses, which focused on types IA_1_, IB, and II. Hence, the commensal group comprised only six strains: 83% type IA_1_ (5/6) and 17% type II (1/6). The most frequently detected lineage among pathogenic strains was again type IA_1_ (10/20), followed by type IB (7/20), and finally type II (3/20), as illustrated in Figure 4. Globally, the analyzed *C. acnes* (*n* = 26) were distributed among five CCs: 31% CC1 (8/26), 27% CC5 and CC4 (both CC counting 7/26 strains), 11.5% CC6 (3/26), and 4% CC72 (1/26). The phylotype analysis revealed a significant difference in the distribution of MLST profiles among groups, with the IA1 type significantly more prevalent in the commensal group compared to the clinical group (*p*-value = 0.179).

**Table 3:** MLST profile of analyzed pathogen and commensal *C. acnes* strains.

ID	*aroE*	*atpD*	*gmk*	*guaA*	*lepA*	*sodA*	*tly*	*camp2*	ST	CC	Type
01	1	1	1	4	1	4	8	6	5	CC5	IB
02	1	1	1	3	1	1	8	6	4	CC4	IA_1_
03	1	1	1	3	1	1	8	6	4	CC4	IA_1_
04	1	1	1	4	1	4	8	6	5	CC5	IB
05	1	1	1	4	1	4	8	6	5	CC5	IB
06	1	1	1	3	1	1	1	1	1	CC1	IA_1_
07	1	1	1	3	1	1	1	1	1	CC1	IA_1_
08	17	4	2	4	2	3	10	10	6	CC6	II
09	17	4	2	4	2	3	10	10	6	CC6	II
10	1	1	1	3	1	1	1	1	1	CC1	IA_1_
11	17	9	2	4	2	3	10	10	25	CC6	II
12	1	1	1	3	1	1	8	6	4	CC4	IA_1_
13	1	1	1	4	1	4	8	6	5	CC5	IB
14	1	1	1	3	1	1	8	6	4	CC4	IA_1_
15	1	1	1	3	1	1	8	6	4	CC4	IA_1_
16	1	1	1	3	1	1	8	6	4	CC4	IA_1_
17	1	1	1	4	1	4	8	21	42	CC5	IB
18	1	1	1	4	1	4	8	6	5	CC5	IB
19	1	1	1	3	1	1	1	1	1	CC1	IA_1_
20	1	1	1	4	1	4	8	21	42	CC5	IB
C_02	17	4	2	4	2	12	10	12	72	CC72	II
C_03	1	1	1	3	1	1	1	1	1	CC1	IA_1_
C_04	1	1	1	3	1	1	8	6	4	CC4	IA_1_
C_05	1	1	1	3	1	1	1	1	1	CC1	IA_1_
C_06	1	1	1	3	1	1	1	1	1	CC1	IA_1_
C_07	5	1	1	3	1	1	1	1	20	CC1	IA_1_
C_09	1	1	1	3	1	1	1	6	105	–	–

#### 3.5.2 BURST analysis

Strains were divided into two groups based on their allelic profiles through the BURST analysis. In particular, for each ST, the numbers of single-locus variants (SLVs), double-locus variants (DLVs), and satellites (SATs) are also reported and summarized in Table 4. In particular, SLVs and DLVs are the sequence types that differ by one or two alleles, respectively. BURST analysis divided the strains into two different groups: Group 1, including types IA_1_ and IB, and Group 2, including type II. The most common genetic variants in Group 1 were ST1 and ST4, each accounting for 27% of the total sample, whereas ST6 accounted for two of the four IB strains reported in Group 2.

**Table 4:** Bacterial phylotypes according to BURST analysis.

ST	Frequency	SLV	DLV	SAT
Group 1				
1	7	2	1	2
4	7	1	2	2
5	5	1	1	3
20	1	1	1	3
42	2	1	0	4
105	1	2	1	2
Group 2				
6	2	1	1	–
25	1	1	0	1
72	1	0	1	1

#### 3.5.3 Phylogenetic tree visualization of phylotypes

**Figure 5:**
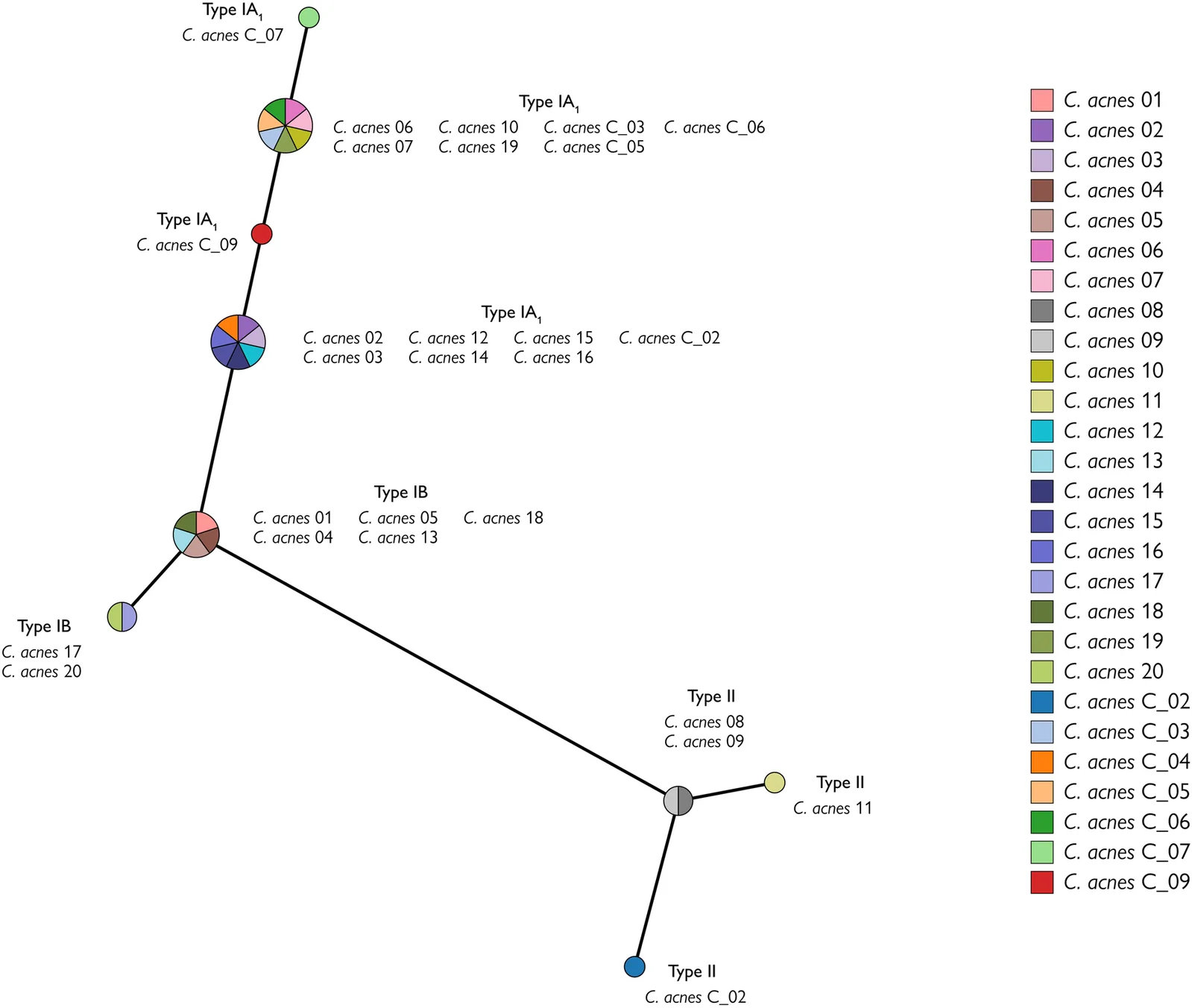
Genetic relationship of the studied strains through GrapeTree software analysis.

**Figure 6:**
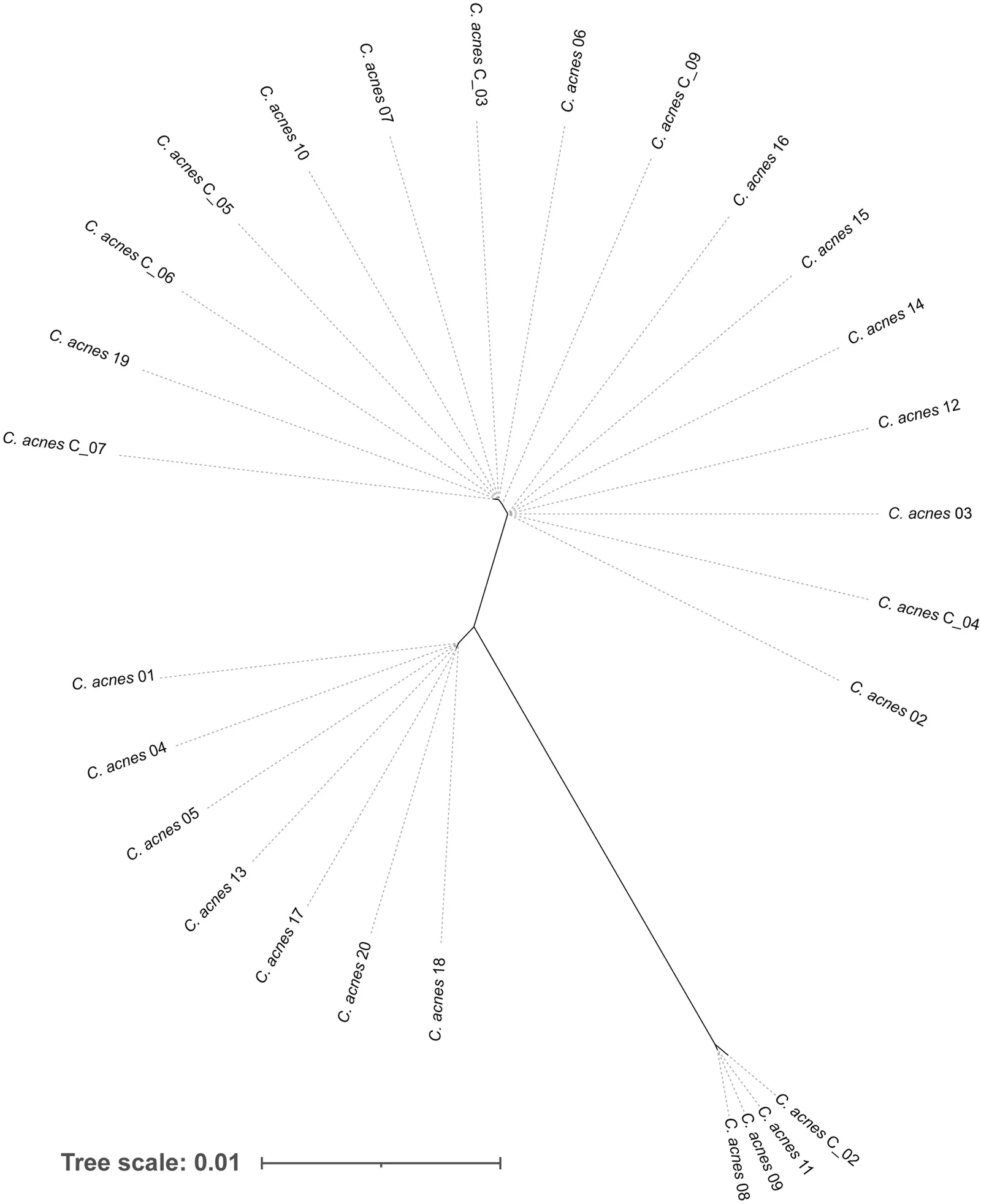
Phylogenetic tree of the studied strains generated with the iTOL online tool.

GrapeTree and iTOL plugins were used to visualize the genomic relationship of the tested pathogens and commensal strains (Figures 5, 6).

As reported in Figure 5, there is a sharp and predictable genetic distance between type IA1/IB and type II strains. However, a clear separation by isolation site was not observed. Indeed, from the reported phylogenetic tree, it is not possible to evidence any difference between pathogens and commensal strains based on the phylogenetic distance/type.

This phenomenon is even more evident in the tree obtained with the iTOL online tool (Figure 6). Here, three tree branches constituted by solid lines are present, representing type IA_1_, IB, and II phylotypes. In particular, type II was the most distant branch, while type IA_1_ and IB strains were closer and characterized by weaker connections among *C. acnes* strains.

### 3.6 Evaluation of biomass formation

**Figure 7:**
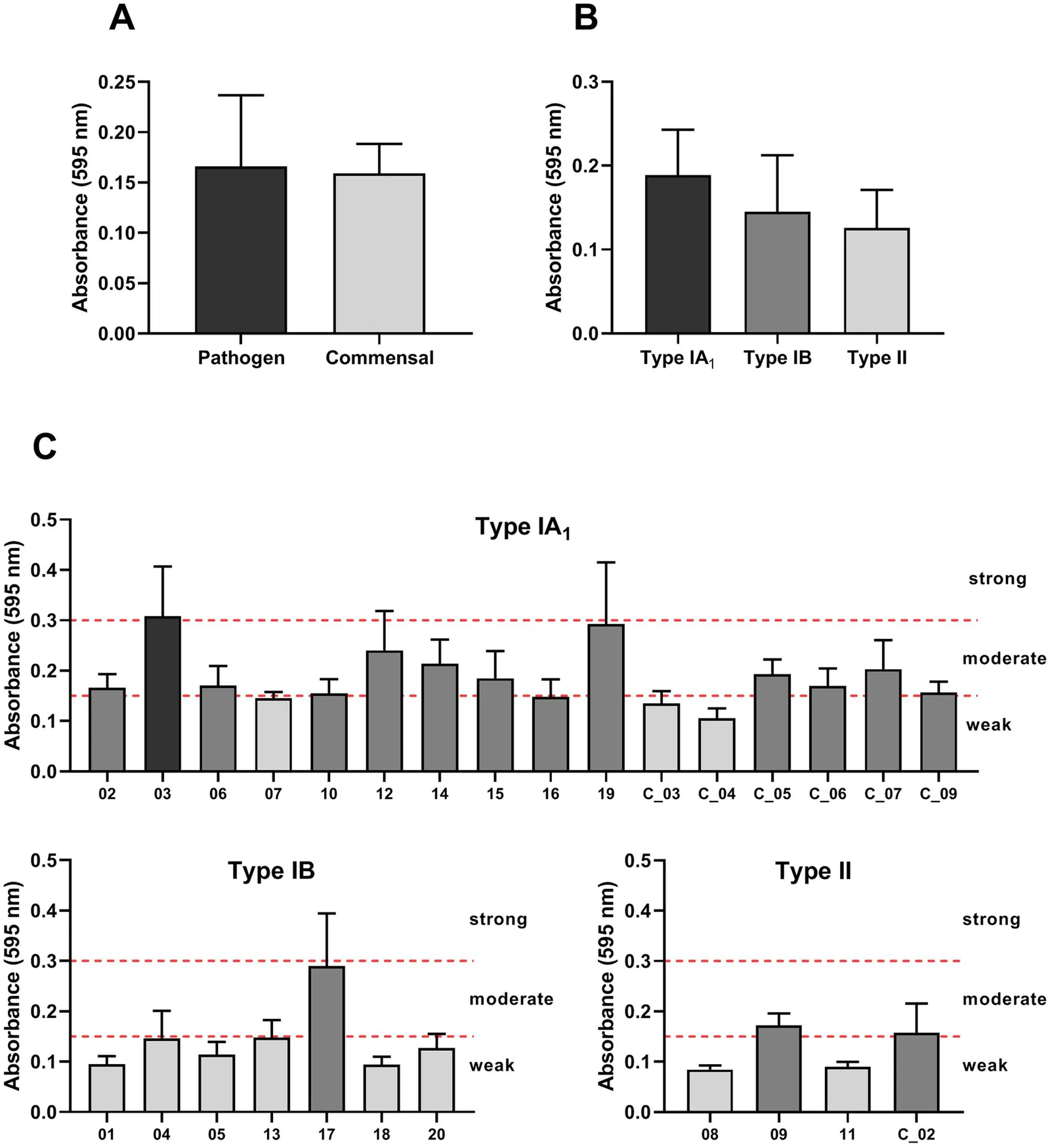
Biofilm biomass production according to the *C. acnes* phylotype. **(A)** Cumulative data for prosthetic joint infection isolates and commensal ones. **(B)** Cumulative data for the different phylotypes. **(C)** Classification of single strains. Data are expressed as absorbance values at 595 nm.

Based on information from the WGS analysis, data from the CV assay were analyzed according to the different *C. acnes* phylotypes (Figure 7). Notably, the variability in biomass formation among the three phenotypes was statistically significant (*p*-value = 0.032): IA_1_ *C. acnes* produced more biofilm than type IB or II strains on average, suggesting that the phylotype might have a greater impact on biomass formation than the isolation site.

## 4 Discussion and conclusions

The increasingly frequent use of implantable biomaterials in the treatment of musculoskeletal pathologies has certainly brought enormous benefits in improving the quality of life of patients who are increasingly elderly. As patients' life expectancy increases, the number of arthroplasties will inevitably grow, accompanied by a rise in complications, such as infections. Indeed, the abiotic surface of implanted medical devices offers contaminants the perfect substrate on which to adhere and form a biofilm (22). Through this elegant mechanism of protection, even commensal bacteria present on human skin and mucosal surfaces, such as coagulase-negative staphylococci and *C. acnes*, might become emerging opportunistic pathogens (23, 24). Furthermore, biofilm confers mechanical protection against immune system attacks and antibiotics, which may not be sufficiently available locally to permeate the slimy matrix (25).

When dealing with *C. acnes*-mediated infections, the diagnostic process can be challenging because of the commensal existence of these bacteria on the skin of healthy participants. In these cases, a positive microbiological culture might be both (i) the result of an accidental contamination or (ii) the effective identification of the etiological agent. Hence, to avoid ambiguity, the isolation of these low-virulence bacteria is usually considered clinically relevant only if the same microorganism is isolated in at least two samples, or in one sample if other major criteria for the diagnosis of PJIs are satisfied (26). However, the definition of the etiological agent is not always straightforward in these cases.

Sometimes, indeed, the *in vitro* culture of *C. acnes* can be challenging, leading to the underestimation of its prevalence in PJIs (27). In fact, the most frequent symptoms of *C. acnes* PJI are persistent joint pain and prosthesis dysfunction. By contrast, fever is typically absent, local inflammatory signs are observed in fewer infections, and serum inflammatory markers such as C-reactive protein or polymorphonuclear leukocytes can be normal or only slightly elevated (28, 29) For these reasons, there is a need to further investigate the role of low-virulence, commensal, emerging pathogens, such as *C. acnes*, in the development of PJI possibly identifying putative hallmarks of subclinical implant-related infections.

This study was performed to acquire phenotypic and genotypic data of different *C. acnes* involved in PJIs and to compare their characteristics with those of other commensal strains. In this work, the clinical isolates analyzed were retrieved from the explanted prosthetic implants and periprosthetic tissues of patients who underwent revision surgery, whereas commensal strains were obtained from the foreheads of healthy volunteers. Unfortunately, the retrospective nature of the study did not permit sampling of commensal specimens from the same patients diagnosed with *C. acnes*-mediated PJIs, representing a limitation of the work. This limitation was taken into account when interpreting the datasets obtained. We considered the possibility that the isolated *C. acnes* could derive from a contamination of the sample rather than the involved pathogen, causing infection. Still, we are prone to exclude it since the isolates used in the study were obtained from at least two of five samples collected during surgery, and they were the only bacteria isolated from patients with a confirmed diagnosis of PJIs.

Several phenotypic characteristics contribute to the ability of *C. acnes* to cause chronic, low-grade infections. First, *C. acnes* expresses several virulence factors that enable it to aggregate and adhere to abiotic surfaces, evade the host immune response, and survive and proliferate in a variety of harsh environments. In our study, all tested bacteria formed biofilms under controlled *in vitro* experimental conditions. According to the classification proposed by Stepanovic and colleagues (15), commensal and clinical isolates were classified as strong, moderate, or weak biofilm producers. Contrary to expectations, *C. acnes* isolated from the skin microbiota produced no less biofilm than the strains retrieved from PJIs. This phenomenon was then confirmed by analysis of bacterial aggregation, which again showed no significant differences contingent on the isolation site. The analysis using confocal scanning laser microscopy revealed the production of GlcNAc-containing polysaccharides and the presence of extracellular DNA in the secreted matrix in both clinical and commensal isolates. All matrix stains were colocalized with the SYTO 9 signal, with intensity proportional to the aggregate size. Unfortunately, it is not possible to discriminate whether the signal is derived from the PNAG produced by bacteria and/or from incorporated hyaluronic or from glucosamine in the proteoglycans found in synovial fluids.

In light of these results, it was hypothesized that genotypic features might drive the different phenotypic behaviors detected in our *in vitro* investigations, rather than the isolation site or their pathogenic nature. To consider this hypothesis, the bacterial genome was sequenced and subsequently analyzed to define the MLST profile. Notably, the phylotypes were not equally abundant. Consistent with data reported in the literature, the IA_1_ type was the most abundant phylotype on both healthy skin and PJI. In particular, 58% of the *C. acnes* evaluated were identified as IA_1_, followed by IB at 27%, and finally type II making up less than 15% of the *C. acnes* analyzed (30). Type III was not detected on any implant-related specimens, as also reported in the study of Sampedro and colleagues (30). *C. acnes* type IA_1_ and IA_2_ are mainly found on skin and their involvement in moderate to severe acne has been reported as well as that of the recently described type IV (31). Differently, types IB and II are mainly described as etiological causes of soft tissue and medical device-related infections (32, 33). Despite that, it was recently reported that phylotypes IA_1_ and IA_2_ were identified in PJI samples (34).

Our data show that type IA_1_ strains produced more biofilms than type IB and II strains. These results are widely confirmed by other recent studies (30, 35). A recent genome-wide association study hypothesized that the ability to cause infections could be associated with specific metabolic pathways or flexibilities rather than with special virulence traits (35), and that strains belonging to the same SLST type can differ in their accessory genome (32). Moreover, the combined effects of microenvironment and genes may influence *C. acnes* function in normal and diseased skin (36).

Based on our findings, which suggest the absence of a correlation between a specific *C. acnes* phenotype and PJI, and supporting literature data, it may be speculated that the pathogenic potential of *C. acnes* depends not exclusively on infection-specific lineages but also on opportunistic access to deep tissues during surgery, the presence of a foreign-body surface, biofilm formation, reduced immune clearance, and the local prosthetic microenvironment. *C. acnes* responsible for PJIs may originate from normal skin microbiota of patients and enter the body during index surgery (27, 37). Once in place, the presence of host fibronectin on the prosthesis surface may enhance the adhesion of *C. acnes*, followed by biofilm production. Moreover, it has been reported that *C. acnes* can internalize into bone-resorbing cell lines, such as osteoclasts and bone-forming cell lines, and that biofilm production by non-infectious *C. acnes* strains is enhanced following internalization by osteoblast-like cells (38). The same authors have shown that tobacco use, known to play a role in inflammatory symptoms in patients with PJIs, influences *C. acnes* persistence in osteoblast-like cells, thus confirming the link between the host and environment in *C. acnes* behavior (39). The production of biofilms and bacterial aggregates, together with cell internalization, may be hypothesized to contribute to evasion of the host inflammatory and immune response, thus allowing bacterial persistence.

However, the limited and unbalanced sampling design, particularly the small number of healthy-skin isolates, is an important limitation of this study, as is the selection of only one isolate from each sample for genomic analysis. Consequently, the genomic differences observed between the groups should not be interpreted as definitive infection-associated traits, as they may also be influenced by incomplete sampling of commensal diversity, phylogenetic lineage, population structure, or clonal relatedness. Nevertheless, the bacterial genomic sequences of both clinical and commensal *C. acnes* strains have been submitted to two different public repositories (PubMLST.org and GeneBank) to enlarge their databases and make data available.

In conclusion, *C. acnes* isolated from PJI and healthy skin share common features that do not allow distinction between commensal and pathogenic isolates, thus suggesting the hypothesis that several external factors influence the pathogenic potential of *C. acnes*. Further studies involving larger, more balanced, and phylogenetically diverse collections of isolates are needed to validate these exploratory findings.

## Data Availability

The datasets presented in this study can be found in online repositories. The names of the repository/repositories and accession number(s) can be found in https://zenodo.org/records/20087400.
